# Improving Attendance: Auditing Clinician’s Adherence to 'Was Not Brought’ (WNB) Policy in Dudley Child and Adolescent Mental Health Service (CAMHS)

**DOI:** 10.1192/bjo.2025.10555

**Published:** 2025-06-20

**Authors:** Bukola Awoyemi, Bolutife Oyatokun, Arif Khan

**Affiliations:** Black Country Healthcare Foundation Trust, Dudley, United Kingdom

## Abstract

**Aims:** Non-attendance (DNA) at appointments wastes NHS resources and can indicate potential risk, making it essential for Trusts to have robust policies to safeguard young people and reduce recurrence. As part of the National Health Service (NHS) process, organisations are required to have a ‘Was Not Brought’ (WNB) policy to address situations where children are not seen. This audit aimed to assess Clinician’s adherence with the Trust’s DNA/WNB policy and reduce WNB rates.

**Methods:** The audit was conducted in two cycles, analysing retrospective data from 50 patient case notes (on RIO) using a pre-determined data tool based on the Trust’s policy at each cycle.

Cycle 1 (August to October 2023) focused on DNA/WNB appointments and included an analysis of 8 patient survey responses, based on the policy 'Protocol for Managing DNA and Cancellations’ in Dudley and Walsall CAMHS.

Following our recommendations, a new policy was drafted, adopted and shared across the Trust.

Cycle 2 (September to November 2024) audited DNA/WNB appointments and incorporated a staff survey (20 responses) based on the new policy 'CAMHS WNB Standard Operating Procedure'.

**Results:** Audit revealed moderate compliance (over 50%) in documenting DNA/WNB appointments, contacting families, and rescheduling appointments across both cycles.

However, compliance was poor (≤40%) in key areas, such as documenting risk assessments, reviewing case notes, attempting to engage with the child, completing lateral checks, sharing rescheduled appointments with GPs/referrers/other agencies, and obtaining SMS consent for appointment reminders.

WNB rates were 8.3% in Cycle 1 and 9% in Cycle 2.

The patient survey revealed that common reasons for WNB included forgetfulness, lack of awareness of the appointment, and illness. Respondents suggested SMS reminders 48 hours before appointments.

The staff survey showed that 45% were unaware of the updated WNB policy, and 30% did not know about the new messaging reminder system and so were not using it.

**Conclusion:** Overall, the Trust policy is generally followed for documenting DNA/WNB appointments and rescheduling; compliance remains poor in areas like risk assessments, engaging with the child, and sharing rescheduled appointments with other agencies.

There is a need for improved documentation and adherence to the WNB policy. The Trust should implement a reminder system to update clinicians on policy changes and prioritize messaging to families to improve appointment attendance.

